# Quantum CZ gates on a single gradient metasurface

**DOI:** 10.1038/s41377-025-01871-5

**Published:** 2025-05-13

**Authors:** Qi Liu, Yu Tian, Zhaohua Tian, Yali Jia, Guixin Li, Xi-Feng Ren, Qihuang Gong, Ying Gu

**Affiliations:** 1 State Key Laboratory of Artificial Microstructure and Mesoscopic Physics, Department of Physics, Peking University, Beijing, 100871 China; 2 Frontiers Science Center for Nano-optoelectronics & Collaborative Innovation Center of Quantum Matter & Beijing Academy of Quantum Information Sciences, Peking University, Beijing, 100871 China; 3 Department of Materials Science and Engineering, Southern University of Science and Technology, Shenzhen, 518055 China; 4 CAS Key Laboratory of Quantum Information, University of Science and Technology of China, Hefei, 230026 China; 5 Hefei National Laboratory, Hefei, 230088 China; 6 Collaborative Innovation Center of Extreme Optics, Shanxi University, Taiyuan, Shanxi 030006 China; 7 Peking University Yangtze Delta Institute of Optoelectronics, Nantong, 226010 China

**Keywords:** Quantum optics, Nanophotonics and plasmonics

## Abstract

For the requirement of quantum photonic integration in on-chip quantum information, we propose a scheme to realize quantum controlled-Z (CZ) gates through single gradient metasurface. Using its parallel beam-splitting feature, i.e., a series of connected beamsplitters with the same splitting ratio, one metasurface can support a polarization encoding CZ gate or path encoding CZ gate, several independent CZ gates, and cascade CZ gates. Taking advantage that the path of output state is locked by the polarization of input state, path encoding CZ gates can efficiently filter out bit-flip errors coming from beam-splitting processes. These CZ gates also have the potential to detect quantum errors and generate high-dimensional entanglement through multi-degree-of-freedom correlation on metasurfaces. By integrating quantum CZ gates into a single metasurface, our results open an avenue for high-density and multifunctional integration of quantum devices.

## Abstract (web summary)

A subwavelength metasurface implements multi-degree-of-freedom quantum CZ gates with cascadable configurations, enabling scalable quantum circuit architectures in compact platforms.

## Graphical abstract

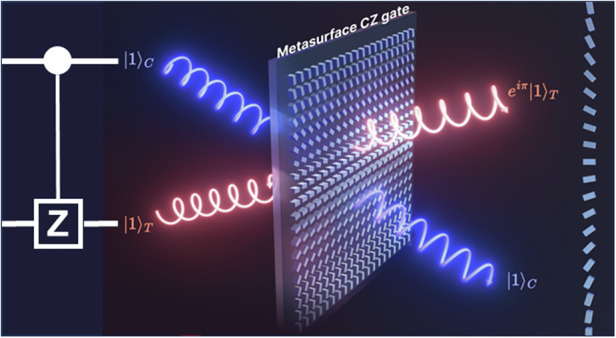

## Introduction

Quantum controlled-NOT (CNOT) or controlled-Z (CZ) gate, conditionally flipping the target qubit or adding a *π* phase by control qubit, is a basic building block of quantum circuit in universal quantum computation^1,2^. Nowadays, the CNOT or CZ gate has been realized in the systems of trapped-ion^3^, superconductor^4^, neutral atom^5^, quantum dot^6^, and linear optics^7,8^. For the requirement of on-chip quantum information processing, linear optical systems become good candidates due to the advantages of long coherence time of photons, high information-capacity of photons^9^, rapid speed of logic operations and simplicity of implementation^10–12^. Using the principles of linear optics, quantum CZ gates were experimentally realized with several identical beamsplitters^13–16^, but their scalability is constrained by size of bulk beamsplitters. Since 2008, on-chip CZ gates have been proposed for both path-encoded^17–19^ and polarization-encoded^20,21^ qubits, with gate dimensions decreased to hundreds of micrometers. With plasmonic structures^22^, novel waveguide coupling techniques^23^, symmetry-breaking^24^ and optical inverse design^25^, size of CZ gates can be further reduced.

Traditionally, one CZ gate is formed by at least three identical beamsplitters. While a quantum device generally needs a set of CZ gates (many beamsplitters), if they are fabricated within a chip, the factors such as asymmetric, crosstalk, and loss, will bring great uncertainty to quantum logic functions. Among these factors, the asymmetry in fabrication^17^ and crosstalk^22^ could lead to unexpected outputs, thus decreasing fidelity of the quantum logical operation. Moreover, coupling photons into and out of a waveguide-based quantum logical device will cause a large loss^23–25^, which makes it inefficient. To reduce the influence of these factors, using an individual micro or nano structure to realize CZ gate is required.

Metasurfaces, emerging as novel planar platforms for light manipulation among multiple degrees of freedom^26–29^, offer a new paradigm for quantum integration^30–32^. Through metasurfaces, the generation^33–35^ and manipulation^36–39^ of high-dimensional entanglement, quantum tomography^40^, quantum imaging^41,42^, quantum sensing^43^, and beam-splitting (BS) functions^44–48^ are proposed, suggesting their potential to integrate quantum devices on a chip. Despite these progresses, the demonstration of fundamental logic operations (such as CNOT or CZ gate) through a single metasurface remains a challenge. According to that gradient metasurface has the peculiarity of parallel BS, i.e., a set of connected beamsplitters with the same splitting ratio^48^, we theoretically propose a scheme to realize quantum CZ gates through single metasurface. Once it is realized, it will become an important basement of quantum logic devices for on-chip quantum information processing.

**Fig. 1:**
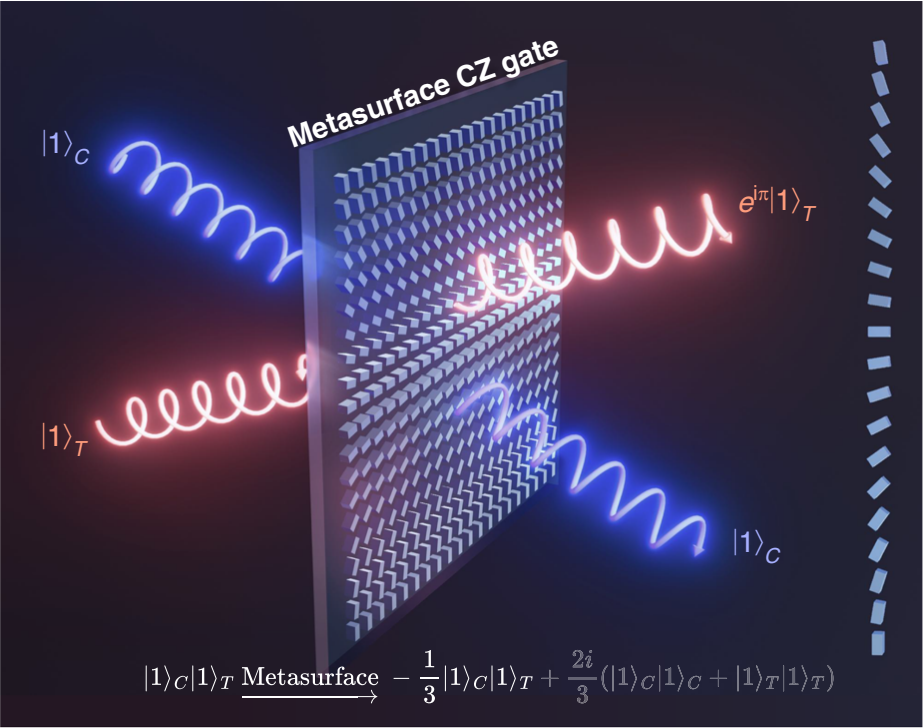
Schematic diagram of polarization-encoded quantum CZ gate through single gradient metasurface

In this work, utilizing three of beamsplitters on a single gradient metasurface, one quantum CZ gate with polarization or path encoding is first demonstrated [Fig. 1]. Choosing another three beamsplitters in the same metasurface, the next independent CZ operation can be simultaneously implemented, and so on. By adding more connected beamsplitters into a CZ operation, cascade CZ gates can be realized. Taking advantage of path-polarization-locked property between input and output state in gradient metasurface, CZ gates can efficiently filter out bit-flip errors coming from BS processes. Additionally, metasurface CZ gates have the potential to detect quantum errors and generate high-dimensional entangled states by leveraging the polarization-path-orbit angular momentum correlation on metasurfaces. By integrating quantum CZ gates into a single metasurface, our results open an avenue for high-density and multifunction quantum logic integration.

## Results

### Polarization-encoding CZ gates

**Fig. 2:**
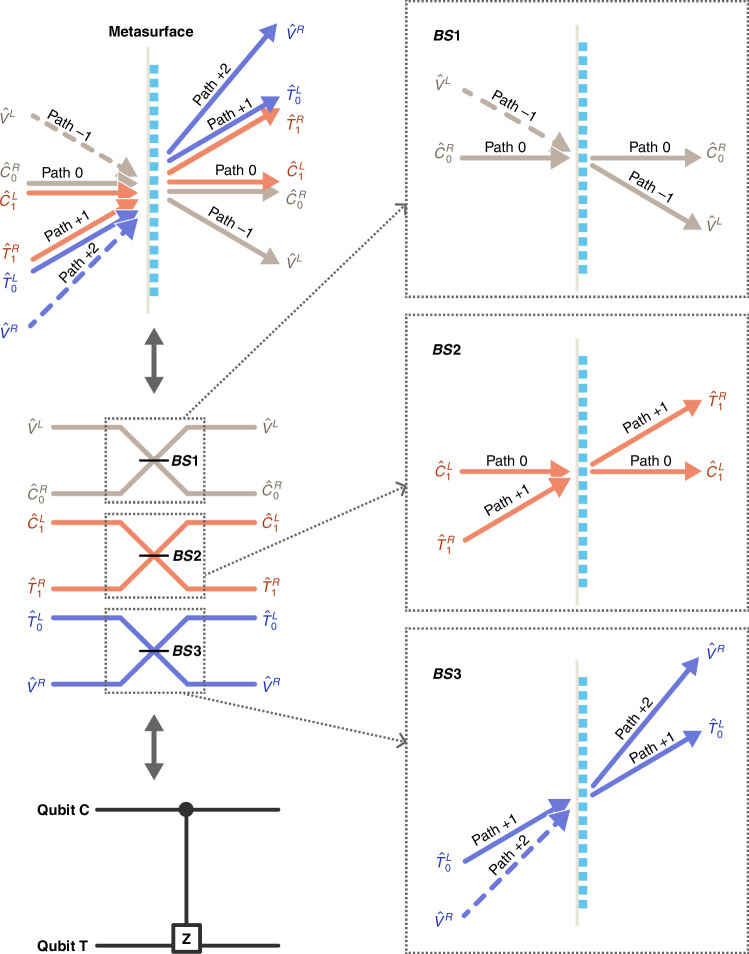
Mechanism of polarization-encoding quantum CZ gate with the help of parallel beam-splitting on the metasurface

The mechanism of metasurface quantum CZ gate is described as follows. The circuit of one CZ operation consists of three identical beamsplitters with 1:2 splitting ratio of power^11,12^. It is recently found that PB phase metasurfaces can act as parallel beamsplitters, i.e., a series of beamsplitters with identical splitting ratios^48^. One can choose three among these splitters to demonstrate a CZ operation. As shown in Fig. 2, two photons are sent to the metasurface with different paths, one of which serves as control qubit and the other as target. The single metasurface performs an equivalent CZ operation through the quantum interference.

The gradient metasurface supports a series of diffraction orders or paths that facilitate parallel BS between polarization modes, but the polarization-path locked relation exists^48^. This implies that for circularly polarized incident light, the zero-order diffraction light has the same polarization state as the input light. While the polarization state of the first-order diffraction light is orthogonal to the input light. Different from previous application where the zero-order transmission light is usually undesired^44,45^, here, we need the zero-order transmission light to carry quantum information. Then both polarization and path can encode qubits. Here, we let qubits be encoded in left-circular (LCP) and right-circular (RCP) polarization of photons [Table 1(a)]. As depicted in Fig. 2, we select two paths (0,+1) for transmitting qubits C and T, paths (−1,+2) are auxiliary. In a waveguide CZ gate, encoding one qubit needs two waveguides^24,25^. While in metasurface CZ gate, thanks to these connected beamsplitters, one polarization qubit can be encoded in a single path using orthogonal polarization modes. Thus, the total path number is reduced from 6 to 4.

**Table 1:** Characteristic of single CZ gate on metasurface

(a) Encoding rules of polarization qubits						
	Control		Target		Auxiliary	
Polarization state	${| R\left.\right\rangle }_{0}$	${| L\left.\right\rangle }_{0}$	${| R\left.\right\rangle }_{+1}$	${| L\left.\right\rangle }_{+1}$	${| R\left.\right\rangle }_{+2}$	${| L\left.\right\rangle }_{-1}$
Encoded qubit state	${| 0\left.\right\rangle }_{C}$	${| 1\left.\right\rangle }_{C}$	${| 1\left.\right\rangle }_{T}$	${| 0\left.\right\rangle }_{T}$	None	None
Mode label	${\hat{C}}_{0}^{R}$	${\hat{C}}_{1}^{L}$	${\hat{T}}_{1}^{R}$	${\hat{T}}_{0}^{L}$	${\hat{V}}^{R}$	${\hat{V}}^{L}$

(b) Truth table of 2-qubit input states		
Input	Output	Post-selection probability
${| 0\left.\right\rangle }_{C}{| 0\left.\right\rangle }_{T}$	${| 0\left.\right\rangle }_{C}{| 0\left.\right\rangle }_{T}$	1/9
${| 0\left.\right\rangle }_{C}{| 1\left.\right\rangle }_{T}$	${| 0\left.\right\rangle }_{C}{| 1\left.\right\rangle }_{T}$	1/9
${| 1\left.\right\rangle }_{C}{| 0\left.\right\rangle }_{T}$	${| 1\left.\right\rangle }_{C}{| 0\left.\right\rangle }_{T}$	1/9
${| 1\left.\right\rangle }_{C}{| 1\left.\right\rangle }_{T}$	$-{| 1\left.\right\rangle }_{C}{| 1\left.\right\rangle }_{T}$	1/9

Using the principle of parallel BS^48^, the transformation relation in Fig. 2 reads

1 $${\left[\begin{array}{c}{\hat{V}}^{R}\\ {\hat{T}}_{0}^{L}\\ {\hat{T}}_{1}^{R}\\ {\hat{C}}_{1}^{L}\\ {\hat{C}}_{0}^{R}\\ {\hat{V}}^{L}\end{array}\right]}_{{\rm{out}}}=\frac{1}{\sqrt{3}}\left[\begin{array}{cccccc}1&\sqrt{2}i&0&0&0&0\\ \sqrt{2}i&1&0&0&0&0\\ 0&0&1&\sqrt{2}i&0&0\\ 0&0&\sqrt{2}i&1&0&0\\ 0&0&0&0&1&\sqrt{2}i\\ 0&0&0&0&\sqrt{2}i&1\end{array}\right]{\left[\begin{array}{c}{\hat{V}}^{R}\\ {\hat{T}}_{0}^{L}\\ {\hat{T}}_{1}^{R}\\ {\hat{C}}_{1}^{L}\\ {\hat{C}}_{0}^{R}\\ {\hat{V}}^{L}\end{array}\right]}_{{\rm{in}}}$$

Here, the qubit and auxiliary modes are related to some polarization modes: $[{\hat{V}}^{R},{\hat{T}}_{0}^{L},{\hat{T}}_{1}^{R},{\hat{C}}_{1}^{L},{\hat{C}}_{0}^{R},{\hat{V}}^{L}]=[{\hat{a}}_{R}(+2),{\hat{a}}_{L}(+1),{\hat{a}}_{R}(+1),{\hat{a}}_{L}(0),{\hat{a}}_{R}(0),{\hat{a}}_{L}(-1)]$, where the annihilation operator ${\hat{a}}_{L}(j)$ or ${\hat{a}}_{R}(j)$ represents LCP or RCP photon occupying path *j*^48^. The function of Eq. (1) is identical to those of waveguides CZ gates^17,19,24,25^.

With above setup, the truth table of CZ operation is shown in Table 1b. When the input state is ${| 1\left.\right\rangle }_{C}{| 1\left.\right\rangle }_{T}$, then according to Eq. (1), the output state becomes,

2 $${| 1\left.\right\rangle }_{C}{| 1\left.\right\rangle }_{T}\mathop{\to}\limits^{{\rm{Metasurface}}}-\frac{1}{3}{| 1\left.\right\rangle }_{C}{| 1\left.\right\rangle }_{T}+\frac{2i}{3}({| 1\left.\right\rangle }_{C}{| 1\left.\right\rangle }_{C}+{| 1\left.\right\rangle }_{T}{| 1\left.\right\rangle }_{T})$$

with a post-selection probability of 1/9 to obtain the state $-{| 1\left.\right\rangle }_{C}{| 1\left.\right\rangle }_{T}$. The quantum interference originating from parallel BS process of the metasurface induces an additional *π* phase shift in Eq. (2), which guarantees the realization of a CZ operation. While for other kinds of input states, corresponding state transformations are shown in Table 1b. These results confirm that a CZ gate has been implemented on single metasurface with a post-selection probability of 1/9, as those reported in refs. ^11,12^. Superior to other linear optical structures^7,16,17^, the quantum CZ gate can be realized within individual metasurface, which is promising to improve integration density.

### Path-encoding CZ gates

**Fig. 3:**
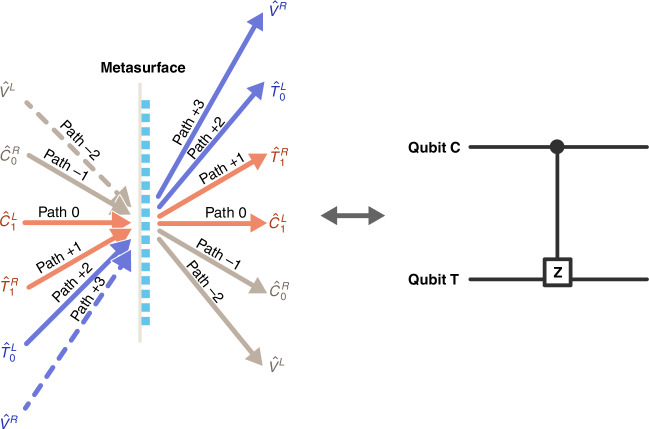
Scheme of path-encoded quantum CZ gate with the help of parallel beam-splitting on the metasurface

Using the same PB metasurface, the parallel BS feature of metasurfaces also enables a path-encoding CZ gate. As illustrated in Fig. 3, a single qubit is carried by two paths. Under this configuration, there are still three BS processes, whose overall transformation matrix remains consistent with Eq. (1). Different from the polarization encoding scheme, the mode correspondence here is $[{\hat{V}}^{R},{\hat{T}}_{0}^{L},{\hat{T}}_{1}^{R},{\hat{C}}_{1}^{L},{\hat{C}}_{0}^{R},{\hat{V}}^{L}]=[{\hat{a}}_{R}(+3),{\hat{a}}_{L}(+2),{\hat{a}}_{R}(+1),{\hat{a}}_{L}(0),{\hat{a}}_{R}(-1),{\hat{a}}_{L}(-2)]$, indicating three independent BS processes. Then, using the same truth table (Table 1b), a CZ operation can be performed. Taking advantage of path-polarization-locked property between input and output state in gradient metasurface, if a path qubit flipping error occurs, whether before entering CZ gates or during propagation within the metasurface, the correlation between path and polarization of output photons will change, preventing normal CZ operation. Thereby, the CZ gate can efficiently filter out bit-flip errors coming from the BS processes. Thus, gradient metasurfaces offer great flexibility in quantum logical operations. Furthermore, metasurfaces can create polarization-path-orbit angular momentum correlation on single photon by adding additional vortex phase modulation^36^. By checking the multi-degree-of-freedom correlation, it is possible to detect quantum errors of qubits with such a metasurface CZ gate. Also, metasurface CZ gates could also enable the creation of high-dimensional entangled states due to the diverse correlation capability.

For a single CZ gate, only two or four adjacent diffraction orders are selected to transmit two qubits. Under the paraxial approximation, the transformation matrix for any two adjacent diffraction orders has the same form as that in Eq. (1)^48^. If we choose other adjacent diffraction orders as transmitting qubits C and T, then on the same metasurface, a new CZ operation can simultaneously exist. Therefore, provided these sets of paths do not interfere with each other, these CZ gates can operate independently. For example, except for paths (0, +1) shown in Fig. 2, we can use paths (−2, −3) to construct another CZ gate. Similarly, more CZ gates are available in the same single metasurface, which means that high-density operations can be integrated within the same piece of metasurface.

### Cascaded CZ gates

**Fig. 4:**
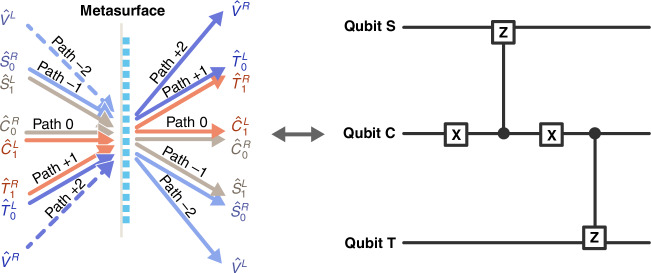
Scheme of a cascade quantum CZ gate with the help of parallel beam-splitting on the metasurface

With the same PB metasurface as above, when two CZ gates supported by single metasurface share a common qubit, they become cascaded. Figure 4 illustrates this cascading configuration, where paths (0, +1) form one CZ gate, and paths (−1, 0) form another, both of which share the path 0 (qubit C). For simplicity, qubits T and C keep the same encoding as Fig. 2, while the third qubit S is encoded as in Table 2a. Then, the transformation relation for input-output qubit modes reads

3 $${\left[\begin{array}{c}{\hat{V}}^{R}\\ {\hat{T}}_{0}^{L}\\ {\hat{T}}_{1}^{R}\\ {\hat{C}}_{1}^{L}\\ {\hat{C}}_{0}^{R}\\ {\hat{S}}_{1}^{L}\\ {\hat{S}}_{0}^{R}\\ {\hat{V}}^{L}\end{array}\right]}_{{\rm{out}}}=\frac{1}{\sqrt{3}}\left[\begin{array}{cccccccc}1&\sqrt{2}i&0&0&0&0&0&0\\ \sqrt{2}i&1&0&0&0&0&0&0\\ 0&0&1&\sqrt{2}i&0&0&0&0\\ 0&0&\sqrt{2}i&1&0&0&0&0\\ 0&0&0&0&1&\sqrt{2}i&0&0\\ 0&0&0&0&\sqrt{2}i&1&0&0\\ 0&0&0&0&0&0&1&\sqrt{2}i\\ 0&0&0&0&0&0&\sqrt{2}i&1\end{array}\right]{\left[\begin{array}{c}{\hat{V}}^{R}\\ {\hat{T}}_{0}^{L}\\ {\hat{T}}_{1}^{R}\\ {\hat{C}}_{1}^{L}\\ {\hat{C}}_{0}^{R}\\ {\hat{S}}_{1}^{L}\\ {\hat{S}}_{0}^{R}\\ {\hat{V}}^{L}\end{array}\right]}_{{\rm{in}}}$$

where the qubit modes and auxiliary modes are $[{\hat{V}}^{R},{\hat{T}}_{0}^{L},{\hat{T}}_{1}^{R},{\hat{C}}_{1}^{L},{\hat{C}}_{0}^{R},{\hat{S}}_{1}^{L},{\hat{S}}_{0}^{R},{\hat{V}}^{L}]=[{\hat{a}}_{R}(+2),{\hat{a}}_{L}(+1),{\hat{a}}_{R}(+1),{\hat{a}}_{L}(0),{\hat{a}}_{R}(0),{\hat{a}}_{L}(-1),{\hat{a}}_{R}(-1),{\hat{a}}_{L}(-2)]$.

**Table 2:** Characteristic of a two-cascaded CZ gate on metasurface

(a) Encoding rules of signal polarization qubit				
	Signal		Auxiliary	
Polarization state	${| R\left.\right\rangle }_{-1}$	${| L\left.\right\rangle }_{-1}$	${| R\left.\right\rangle }_{+2}$	${| L\left.\right\rangle }_{-2}$
Encoded qubit state	${| 0\left.\right\rangle }_{S}$	${| 1\left.\right\rangle }_{S}$	None	None
Mode label	${\hat{S}}_{0}^{R}$	${\hat{S}}_{1}^{L}$	${\hat{V}}^{R}$	${\hat{V}}^{L}$

(b) Truth table of 3-qubit input states				
S&T in standard basis		S&T in Hadamard basis		
Input	Output	Input	Output	Post-selection probability
${| 0\left.\right\rangle }_{C}{| 0\left.\right\rangle }_{S}{| 0\left.\right\rangle }_{T}$	${| 0\left.\right\rangle }_{C}{| 0\left.\right\rangle }_{S}{| 0\left.\right\rangle }_{T}$	${| 0\left.\right\rangle }_{C}{| +\left.\right\rangle }_{S}{| +\left.\right\rangle }_{T}$	${| 0\left.\right\rangle }_{C}{| -\left.\right\rangle }_{S}{| +\left.\right\rangle }_{T}$	1/27
${| 0\left.\right\rangle }_{C}{| 0\left.\right\rangle }_{S}{| 1\left.\right\rangle }_{T}$	${| 0\left.\right\rangle }_{C}{| 0\left.\right\rangle }_{S}{| 1\left.\right\rangle }_{T}$	${| 0\left.\right\rangle }_{C}{| +\left.\right\rangle }_{S}{| -\left.\right\rangle }_{T}$	${| 0\left.\right\rangle }_{C}{| -\left.\right\rangle }_{S}{| -\left.\right\rangle }_{T}$	1/27
${| 0\left.\right\rangle }_{C}{| 1\left.\right\rangle }_{S}{| 0\left.\right\rangle }_{T}$	$-{| 0\left.\right\rangle }_{C}{| 1\left.\right\rangle }_{S}{| 0\left.\right\rangle }_{T}$	${| 0\left.\right\rangle }_{C}{| -\left.\right\rangle }_{S}{| +\left.\right\rangle }_{T}$	${| 0\left.\right\rangle }_{C}{| +\left.\right\rangle }_{S}{| +\left.\right\rangle }_{T}$	1/27
${| 0\left.\right\rangle }_{C}{| 1\left.\right\rangle }_{S}{| 1\left.\right\rangle }_{T}$	$-{| 0\left.\right\rangle }_{C}{| 1\left.\right\rangle }_{S}{| 1\left.\right\rangle }_{T}$	${| 0\left.\right\rangle }_{C}{| -\left.\right\rangle }_{S}{| -\left.\right\rangle }_{T}$	${| 0\left.\right\rangle }_{C}{| +\left.\right\rangle }_{S}{| -\left.\right\rangle }_{T}$	1/27
${| 1\left.\right\rangle }_{C}{| 0\left.\right\rangle }_{S}{| 0\left.\right\rangle }_{T}$	${| 1\left.\right\rangle }_{C}{| 0\left.\right\rangle }_{S}{| 0\left.\right\rangle }_{T}$	${| 1\left.\right\rangle }_{C}{| +\left.\right\rangle }_{S}{| +\left.\right\rangle }_{T}$	${| 1\left.\right\rangle }_{C}{| +\left.\right\rangle }_{S}{| -\left.\right\rangle }_{T}$	1/27
${| 1\left.\right\rangle }_{C}{| 0\left.\right\rangle }_{S}{| 1\left.\right\rangle }_{T}$	$-{| 1\left.\right\rangle }_{C}{| 0\left.\right\rangle }_{S}{| 1\left.\right\rangle }_{T}$	${| 1\left.\right\rangle }_{C}{| +\left.\right\rangle }_{S}{| -\left.\right\rangle }_{T}$	${| 1\left.\right\rangle }_{C}{| +\left.\right\rangle }_{S}{| +\left.\right\rangle }_{T}$	1/27
${| 1\left.\right\rangle }_{C}{| 1\left.\right\rangle }_{S}{| 0\left.\right\rangle }_{T}$	${| 1\left.\right\rangle }_{C}{| 1\left.\right\rangle }_{S}{| 0\left.\right\rangle }_{T}$	${| 1\left.\right\rangle }_{C}{| -\left.\right\rangle }_{S}{| +\left.\right\rangle }_{T}$	${| 1\left.\right\rangle }_{C}{| -\left.\right\rangle }_{S}{| -\left.\right\rangle }_{T}$	1/27
${| 1\left.\right\rangle }_{C}{| 1\left.\right\rangle }_{S}{| 1\left.\right\rangle }_{T}$	$-{| 1\left.\right\rangle }_{C}{| 1\left.\right\rangle }_{S}{| 1\left.\right\rangle }_{T}$	${| 1\left.\right\rangle }_{C}{| -\left.\right\rangle }_{S}{| -\left.\right\rangle }_{T}$	${| 1\left.\right\rangle }_{C}{| -\left.\right\rangle }_{S}{| +\left.\right\rangle }_{T}$	1/27

According to Eq. (3), we analyze the transformations for different input qubits. Figure 4b shows the equivalent quantum circuit of the cascaded CZ gates, the additional X gates mean qubits S and T are controlled by different logical states of qubit C. As shown in the first two columns of truth Table 2b, when qubit C is ${| 0\left.\right\rangle }_{C}$, qubit T is unaffected and qubit S acquires a phase shift when it is in state ${| 1\left.\right\rangle }_{S}$. Conversely, when qubit C is ${| 1\left.\right\rangle }_{C}$, qubit S remains unchanged and qubit T acquires a phase shift when it is ${| 1\left.\right\rangle }_{T}$. The quantum logical function is more obvious when expressing qubits S and T in Hadamard basis ${| \pm \left.\right\rangle }_{S,T}=({| 0\left.\right\rangle }_{S,T}\pm {| 1\left.\right\rangle }_{S,T})/\sqrt{2}$ [the third and fourth columns of truth Table 2(b)], where qubit S/T flips when qubit C is ${| 0\left.\right\rangle }_{C}$/${| 1\left.\right\rangle }_{C}$. Repeating this process, in principle, one metasurface can cascade more CZ gates. Thus, the same metasurface not only enables independent CZ gates, but also allows for the cascaded CZ gates, which is favorable for multifunctional quantum integration.

A single CZ gate can entangle two qubits^2^, while a quantum circuit with cascade CZ gates can establish multiqubit entanglement. For instance, if the input qubit state is set as ${| +\left.\right\rangle }_{S}{| +\left.\right\rangle }_{C}{| +\left.\right\rangle }_{T}$, where $| \pm \left.\right\rangle =(| 0\left.\right\rangle \pm | 1\left.\right\rangle )/\sqrt{2}$ (all three photons in horizontal polarization), then we can use the metasurface configuration depicted in Fig. 4 to prepare entangled state. According to Eq. (3), if the quantum circuit operation is successfully executed, the output state will evolve to $({| +\left.\right\rangle }_{S}{| 1\left.\right\rangle }_{C}{| -\left.\right\rangle }_{T}+{| -\left.\right\rangle }_{S}{| 0\left.\right\rangle }_{C}{| +\left.\right\rangle }_{T})/\sqrt{2}$, which is a three-qubit GHZ entangled state. The cascade CZ gates on the metasurface provide a convenient method for preparing multiqubit entangled states, which can be also utilized for multiqubit entanglement swapping^49,50^.

## Discussion

The proposed quantum CZ gates based on metasurface have advantages compared to those based on waveguides^22–25^. Firstly, metasurfaces offer more precise control on polarization, enabling the degree of circular polarization to remain nearly unity before and after beam-splitting^45^. As a result, compared to polarization-encoding quantum gates based on hybrid waveguides^22^, metasurfaces can effectively decrease errors resulting from crosstalk. Secondly, metasurfaces are also capable of efficiently transmitting photons. By employing special designs, the transmission efficiency can reach up to approximately 96% in experiments^51^. There is no need to use additional devices to derive photon from metasurface quantum gates, so it is more efficient for photon detection than a waveguide-based on-chip quantum gate that suffers from coupler loss^23–25^. Hence, it is expected that metasurface-based quantum CZ gates will achieve a high operational fidelity and efficiency.

To verify above architecture for CZ gates, we design a metasurface using full-wave simulation (see Supplementary Section S1). The simulation results show that the designed metasurface provides a BS ratio of about 1.99 for normally incident light with circular polarization. When the incident angle changes from −15° to +15°, the splitting ratio is almost unchanged, while the total transmission efficiency exceeds 95%. As a result, the metasurface can support up to 6 parallel 2 × 2 BS processes which are connected. Furthermore, within the same range of the incident angle, the degree of circular polarization of output light keeps above 99.3% (absolute value), which means the qubit can be safely encoded in circular polarized states (see Supplementary Section S2). Then, we check the performance of two quantum CZ gates on the metasurface. The simulated truth tables agree well with the ideal results in Tables 1, 2, while the fidelity of the prepared entangled state is higher than 99% (see Supplementary Section S3). The slight deviation mainly comes from the small BS ratio error and phase error of the designed metasurface. Therefore, the designed metasurface structure has a high performance in quantum logic operation.

Finally, we discuss the experimental feasibility of the scheme. A high-efficiency metasurface can be fabricated using electron beam lithography with high-index dielectric materials, such as amorphous silicon^44^, silicon nitride^47^ for near-infrared wavelengths or titanium dioxide^45^ for visible light. Recent researches have shown that the transmission efficiency of dielectric metasurfaces can reach as high as 96% in experiment^44,51–53^, so the post-selection probability of the quantum gate should not decrease too much. The fidelity of CZ gate is primarily affected by splitting ratios. Due to the imperfections in the fabrication of the metasurface, the zero-order transmission may be increased, which will lead to splitting ratios deviate 1:2 slightly. Thus, the fidelity of the CZ gate will decrease to some degree, as the post-selection probabilities become unequal for different input states. However, if the error in splitting ratios can be kept within 5%, the fidelity of CZ gate is still higher than 90%, which have been verified by theory^11^ and experiments in waveguide system^17,23–25^. So, in practical situations, it is necessary to correct the design size of the metasurface unit cell by calibrating the fabrication deviation to make the error of splitting ratio as small as possible. Thanks to current micro-/nanofabrication technology, the splitting ratios error of metasurfaces can be controlled within 5%^45,47^. Another challenge in experimental implement is the alignment of the beam paths. For the metasurface we designed, it is fairly challenging to precisely manage the relative angle between two adjacent paths (~5^∘^). Anyhow, our scheme is likely to be realized experimentally in the near future.

To summarize, on a single gradient metasurface, we theoretically demonstrated a polarization encoding CZ gate, a path encoding CZ gate, several independent CZ gates, and cascaded CZ gates. The path encoding CZ gates can efficiently filter out bit-flip errors due to path-polarization-locked property of gradient metasurface. Furthermore, metasurface CZ gates may enable detection of quantum errors and preparation of high-dimensional entangled states through the polarization-path-orbit angular momentum correlation on metasurfaces. By integrating several quantum CZ gates into a single metasurface, various quantum operations can be performed with only a piece of metasurface. Based on the same principle, multi-qubit logical operations could be executed on single metasurface. Thus, this work paves the way for high-density, multifunctional integration of quantum logic devices on metasurfaces, with potential applications in on-chip quantum information processing.

## Materials and methods

### Design of metasurface for CZ operations

The quantum CZ gate operation discussed in this work is realized with a geometric phase gradient metasurface. The unit cells of such a metasurface exhibit local birefringence response. We choose an operating wavelength of 1550 nm and use nanofin structures on a glass substrate (with a refractive index of 1.5^43^) as the structural units. The nanofin structures are made of amorphous silicon (with a refractive index of 3.344^43^), with dimensions of 389 nm × 219 nm × 830 nm. The centers of adjacent structural units are spaced 667 nm apart. Under this set of parameters, the metasurface can achieve a transmission efficiency close to 1 and a cross-polarization conversion efficiency of approximately 2/3. To achieve beam splitting, a geometric phase gradient is introduced, with 26 structural units stitched together to form a period. The adjacent structural units are rotated successively by an angle of 2*π*/26. The geometric phase gradient causes the cross-polarization conversion light to deflect to the +1 or −1 diffraction order, with the 0th order diffraction energy to 1st order diffraction energy ratio approaching 1: 2, and the relative phase difference approaching *π*/2. The specific beam splitting transformation matrix can be obtained through finite element method simulation (commercial COMSOL software). Details of the simulation are provided in the supplementary Section S1 and S2. It is flexible to design metasurface CZ gates with other low-loss, high-index materials, like titanium dioxide^54–56^ and gallium nitride^57,58^. The main differences among these material platforms lie in the geometric size of the designed unit cells and the working wavelength.

In principle, it is possible to realize a quantum CZ gate with other kinds of metasurfaces rather than PB phase metasurfaces. For polarization-encoding CZ gate, the crucial aspect is to achieve the multi-port beam-splitting response similar to that in equation (1). So, a metasurface with polarization-dependent amplitude and phase modulation may help to achieve this goal, such as the approach of anisotropic propagation phases^44,45^. Another possible design to achieve polarization-encoding CZ gate might be metasurfaces composed of freeform meta-atom structures. Using the photonics inverse design technique^59,60^, one can start with the target multi-port beam-splitting response in equation (1), then design a shape of meta-atom by topological optimization.

### Quantum state transformation on metasurface

The quantum parallel beam splitting process in metasurfaces can be described by the effective Hamiltonian^48^${\hat{H}}_{{\rm{eff}}}={\sum }_{j}{\hat{H}}_{j}$, where ${\hat{H}}_{j}=-\hslash g[{\hat{a}}_{L}^{{\rm{in\dagger }}}(j){\hat{a}}_{R}^{{\rm{in}}}(j+1)+{\hat{a}}_{L}^{{\rm{in}}}(j){\hat{a}}_{R}^{{\rm{in\dagger }}}(j+1)]$ characterizes the beamsplitter-type interaction between the LCP mode at diffraction order *j* and the RCP mode at diffraction order *j* + 1. Consequently, the time evolution operator is given by $\hat{S}(t)=\exp [-i{\hat{H}}_{{\rm{eff}}}t/\hslash ]$, where the effective interaction time satisfies $gt=\arccos (1/\sqrt{3})$, corresponding to the required 1:2 beam splitting ratio. Thus, given an input quantum state $| {\psi }_{{\rm{in}}}\left.\right\rangle$, the output state after passing through the metasurface is $| {\psi }_{{\rm{out}}}\left.\right\rangle =\hat{S}(t)| {\psi }_{{\rm{in}}}\left.\right\rangle$. The truth table of the metasurface CZ gate is obtained by analyzing the output state of the encoded logical states $| 00\left.\right\rangle ,| 01\left.\right\rangle ,| 10\left.\right\rangle ,| 11\left.\right\rangle$. The exact truth table and quantum state transformation of the designed metasurface CZ gates are provided in the supplementary Section S3.

## Supplementary information

Supplemental Material for Quantum CZ gates on a single gradient metasurface

## Data Availability

All data needed to evaluate the conclusions in this study are presented in the main text and in the supplementary materials.
