# Supplementary material for: Quantum CZ gates on a single gradient metasurface
Source: Light Sci Appl. 2025 May 13;14:193. doi: 10.1038/s41377-025-01871-5 (PMC12075864; doi:10.1038/s41377-025-01871-5)
Supplement: Supplementary file 1 — Supplemental Material for Quantum CZ gates on a single gradient metasurface [file 41377_2025_1871_MOESM1_ESM.pdf]

Supplemental Material for

Quantum CZ gates on a single gradient metasurface

Qi Liu[1,2], Yu Tian[1,2], Zhaohua Tian[1], Yali Jia[1], Guixin Li[3],
Xi-Feng Ren[4,5], Qihuang Gong[1,2,5,6,7], and Ying Gu[1,2,5,6,7*]

[1]*State Key Laboratory of Artificial Microstructure and Mesoscopic Physics, Department of Physics, Peking University, Beijing 100871, China*

[2]*Frontiers Science Center for Nano-optoelectronics & Collaborative Innovation Center of Quantum Matter & Beijing Academy of Quantum Information Sciences, Peking University, Beijing 100871, China*

[3]*Department of Materials Science and Engineering, Southern University of Science and Technology, Shenzhen 518055, China*

[4]*CAS Key Laboratory of Quantum Information, University of Science and Technology of China, Hefei 230026, China*

[5]*Hefei National Laboratory, Hefei 230088, China*

[6]*Collaborative Innovation Center of Extreme Optics, Shanxi University, Taiyuan, Shanxi 030006, China*

[7]*Peking University Yangtze Delta Institute of Optoelectronics, Nantong 226010, China*

* ygu@pku.edu.cn

S1. DESIGN OF METASURFACE

Here, we present a specific metasurface design to implement the quantum CZ gate scheme proposed in the main text. We employ linear birefringent nanostructures to construct the requisite metasurface. The geometric structure of the structural unit is shown in Fig. S1(a), featuring a silicon nanofin structure on a SiO_2 substrate. The silicon nanofin has a height of 830 nm, with the length (l) and width (w) to be determined. The structural units are periodically arranged in the x and y directions with a period of 667 nm. Such a nanostructure acts as a local waveplate, with the corresponding Jones matrix [1, 2]

$$J = \begin{pmatrix} t_{xx}e^{i\phi_x} & 0 \\ 0 & t_{yy}e^{i\phi_y} \end{pmatrix} = t_{xx}e^{i\phi_x}|x\rangle\langle x| + t_{yy}e^{i\phi_y}|y\rangle\langle y| \tag{S1}$$

where $|x\rangle, |y\rangle$ are unit Jones vectors of x-polarized and y-polarized light. We have assumed that the major and minor axes of the nanofin structure are aligned along the x and y directions, respectively. The equation (S1) indicates that x-polarized or y-polarized light maintains its polarization after passing through the nanostructure, accumulating phase shifts of ϕ_x or ϕ_y, with transmission efficiencies of t_{xx}^2 or t_{yy}^2, respectively. However, light with other polarization states will experience a change in polarization upon passing through this structure. In our quantum CZ gate scheme, the operating polarization state is circularly polarized. When expanded using circular polarization basis vectors, the Jones matrix of the nanofin is given by

$$J = \frac{t_{xx}e^{i\phi_x} + t_{yy}e^{i\phi_y}}{2}(|L\rangle\langle L| + |R\rangle\langle R|) + \frac{t_{xx}e^{i\phi_x} - t_{yy}e^{i\phi_y}}{2}(|L\rangle\langle R| + |R\rangle\langle L|) \tag{S2}$$

Upon passing through the structure, part of the circularly polarized light undergoes co-polarized conversion ($|L\rangle \to |L\rangle, |R\rangle \to |R\rangle$), while the other part undergoes cross-polarized conversion ($|L\rangle \to |R\rangle, |R\rangle \to |L\rangle$). The co-polarized and cross-polarized transmission ratios are

$$T_{\text{Co}} = \frac{1}{4}|t_{xx}e^{i\phi_x} + t_{yy}e^{i\phi_y}|^2 \tag{S3a}$$

$$T_{\text{Cross}} = \frac{1}{4}|t_{xx}e^{i\phi_x} - t_{yy}e^{i\phi_y}|^2 \tag{S3b}$$

Employing the geometric phase method enables the separation of co-polarized and cross-polarized light, achieving beam splitting. By changing the length (l) and width (w) of

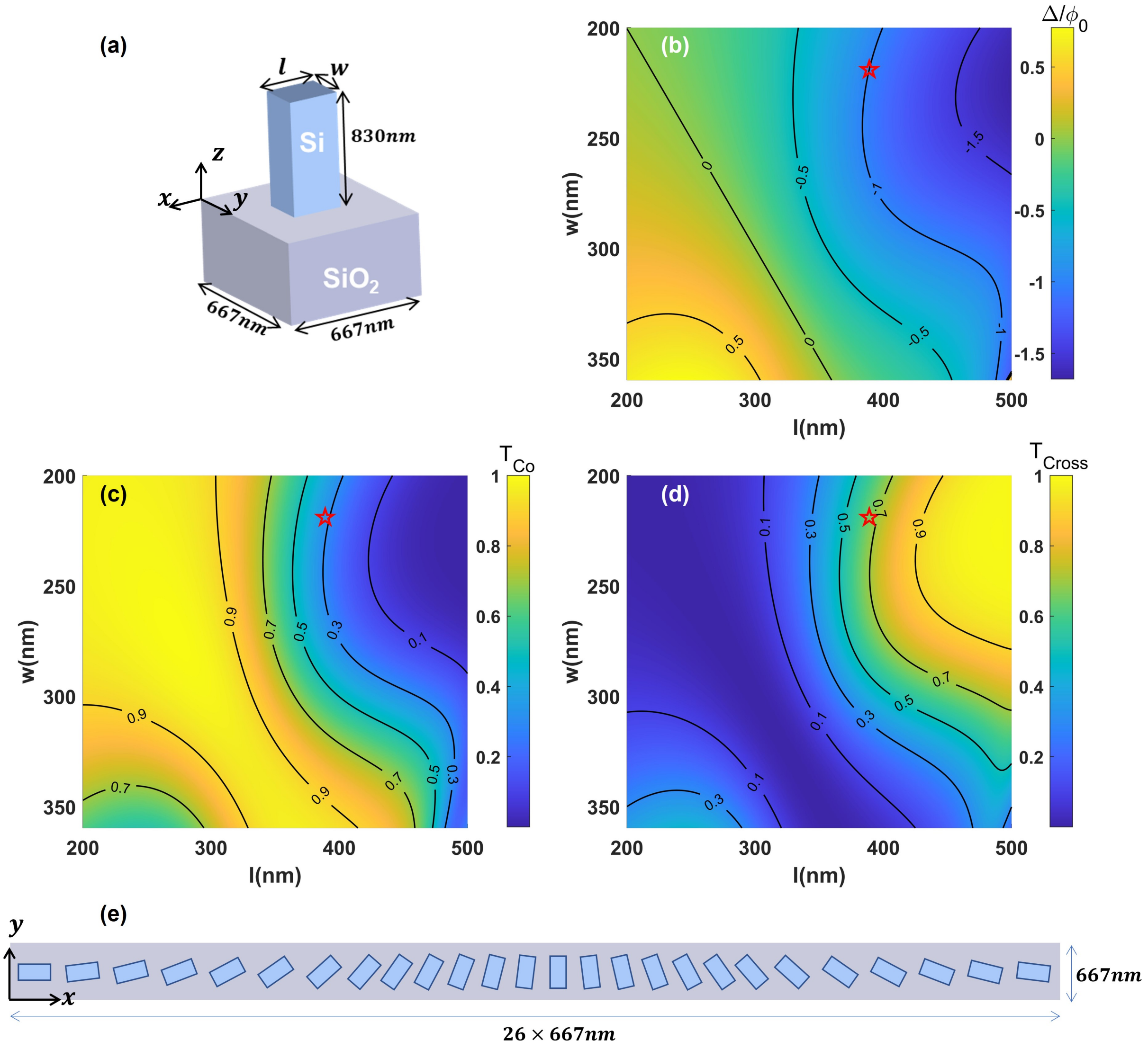

Figure S1. Simulated birefringent responses of individual unit cell in metasurface. (a) The geometry parameters of a metasurface unit cell. (b) The simulated birefringent phase difference; (c) co-polarization transmission efficiency; (d) cross-polarization transmission efficiency of the unit cell as nanofin length l and width w vary. (e) Top view of one period of the designed metasurface. Here, the working wavelength is $\lambda = 1550$ nm, the refractive index of silicon nanofin and SiO_2 substrate are $n_{\mathrm{Si}} = 3.344 + 5.1 \times 10^{-6} i$, $n_{\mathrm{SiO_2}} = 1.5$, respectively. $\phi_0 = \arccos(1/\sqrt{3})$ in (b). The red star marks the designed parameters $(l, w) = (389\ \mathrm{nm}, 219\ \mathrm{nm})$ that meet the requirement for quantum CZ gate.

the nanofin, the beam splitting ratio can be adjusted [1, 2]. When $t_{xx} \approx t_{yy}$, the beam splitting ratio is determined by the birefringence phase difference $\Delta = (\phi_x - \phi_y)/2$. In our scheme, a beam splitting ratio of $T_{\mathrm{Cross}} : T_{\mathrm{Co}} = 2 : 1$ is desired, corresponding to $\Delta = \pm \arccos(1/\sqrt{3})$. The rigorous coupled-wave analysis (RCWA) [4] or finite-element method simulation (commercial COMSOL software) can be used to calculate T_{Cross}, T_{Co}, and Δ. Scanning the parameters of the nanofin's length (l) and width (w) can identify suitable dimensions that meet the aforementioned criteria.

Figures S1(b)-(d) present the simulation results of the parameter sweeping, with the working wavelength set at 1550 nm and the refractive indices of silicon and SiO_2 being $n_{\mathrm{Si}} = 3.344 + 5.1 \times 10^{-6} i$ and $n_{\mathrm{SiO_2}} = 1.5$ [3], respectively. The swept parameter range are from 200 nm to 500 nm for length (l) and from 200 nm to 360 nm for width (w). Within this range, a suitable set of parameters was identified as $l = 389$ nm and $w = 219$ nm, yielding $T_{\mathrm{Cross}} \approx 0.651$, $T_{\mathrm{Co}} \approx 0.326$, and $\Delta \approx -\arccos(1/\sqrt{3})$, marked by the red star in Figs. S1(b)-(d). The ratio $T_{\mathrm{Cross}}/T_{\mathrm{Co}}$ is approximately 1.99, with an overall transmission efficiency exceeding 97%.

With the desired unit cell obtained, the metasurface for implementing the quantum CZ gate can be constructed using the geometric phase method. Figure S1(e) illustrates the distribution of unit cells within a period of the metasurface. A period consists of 26 unit cells, each rotated at different angles. The rotation angle of the unit cells increases sequentially with position within a period, totaling a 180-degree rotation. The cross-polarized light experiences a linear phase gradient $k_{\mathrm{G}} = 2\pi/\Lambda$, with $\Lambda = 26 \times 667$ nm. The LCP and RCP light experience opposite phase gradients during the cross-polarization process, thus achieving beam splitting [3].

S2. VALIDATION OF PARALLEL BEAM SPLITTING

This section validates the parallel beam splitting response of the metasurface designed in the previous section. To this end, full-wave simulations are performed for a plane wave incident on a metasurface with periodic boundary conditions. For the designed metasurface with a period of $\Lambda = 26 \times 667$ nm along x direction, the angles of the various diffraction orders are approximately $\cdots, -10^\circ, -5^\circ, 0^\circ, +5^\circ, +10^\circ, \cdots$, and so on, according to diffraction theory. We define the paths that light takes before/after passing through the metasurface

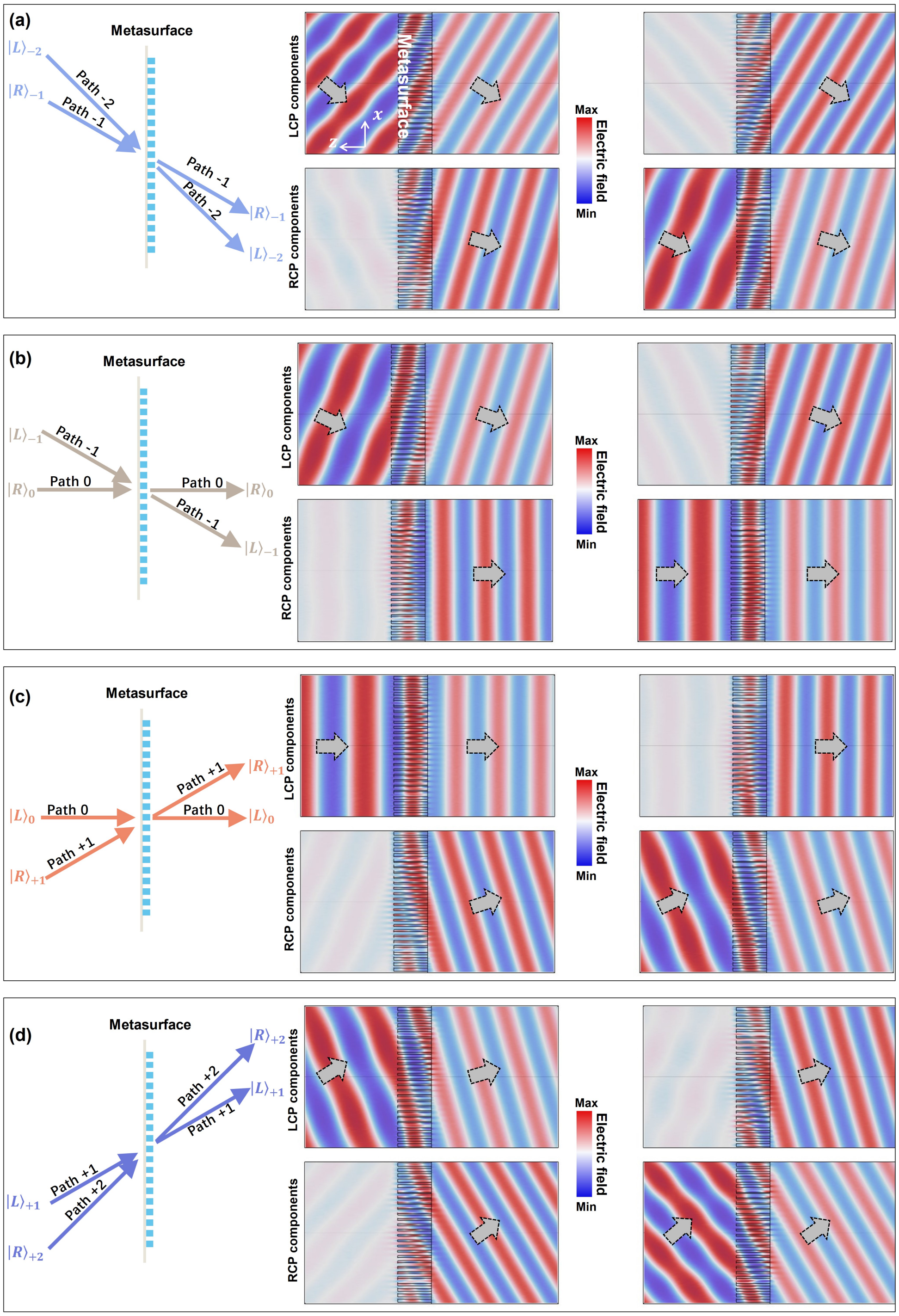

Figure S2. Parallel beam splitting response of the designed metasurface. (a) Schematic of the first circular polarization beam splitting processes (left side), with simulated LCP and RCP electric field distributions decomposed and shown for both LCP (the middle part) and RCP (right side) incidences. (b), (c), and (d) Display electric field distributions for the second, third, and fourth splitting processes, respectively, with similar decompositions. Field distributions are within one period, and the vertical axis is compressed by 5 times for clarity.

from these angles as ..., path+2, path+1, path0, path−1, path−2, and so on, as mentioned in the main text.

Figures S2(a)-(d) display the full-wave simulation results for the four sets of parallel circularly polarized beam splitting processes involved in the main text. On the left side of Fig. S2(a) is the first set of 2×2 beam splitting process, namely the process of LCP light incident from path−2 and RCP light incident from path−1. In the center of Fig. S2(a) is the electromagnetic field distribution when LCP light is incident from path−2, while on the right side is the distribution when RCP light is incident from path−1. Here, the LCP and RCP components of the total electric field are decomposed, showing that after passing through the metasurface, the path−2 remains LCP, and the path−1 remains RCP. Both co-polarized and cross-polarized conversions occur upon incidence from the two paths, with the cross-polarized light deflected into different paths, clearly demonstrating the distinct intensities of co-polarized and cross-polarized light. Figures S2(b)-(d) present the other three sets of beam splitting processes, with similar results. These four sets of beam splitting processes are relatively independent and can occur in parallel, forming the basis of the main text's scheme.

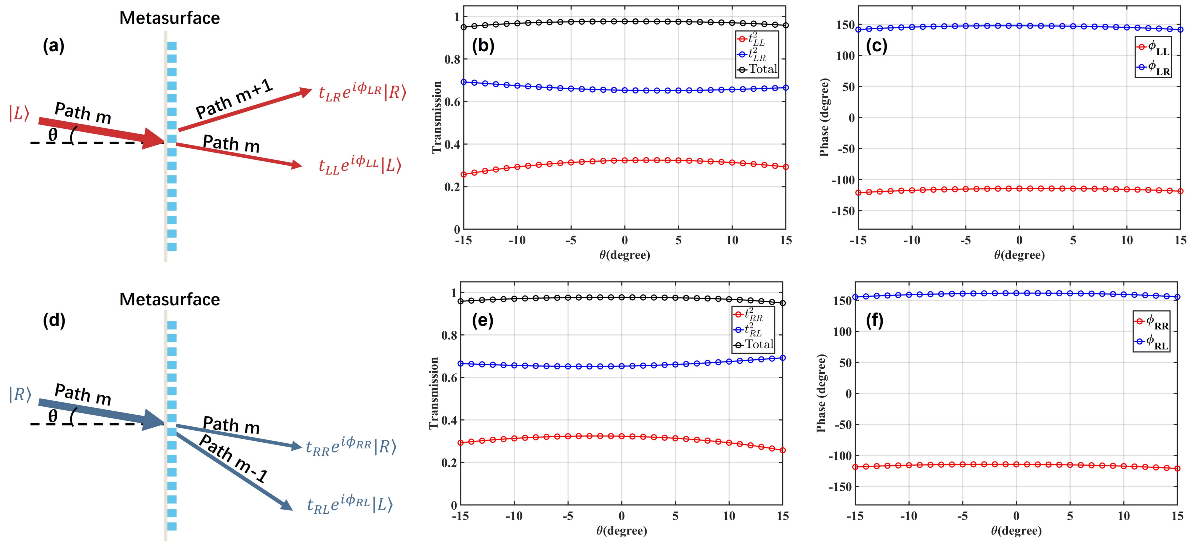

Figure S3. Angular dispersion of beam splitting response for the designed metasurface. (a) Schematic of beam splitting processes for LCP light oblique incidences. (b) Transmission efficiencies and (c) phases of the splitting light in the case of (a). (d)(e)(f) are similar to (a)(b)(c) but for RCP light oblique incidences.

We further calculated the beam splitting ratio and phase for the parallel beam splitting process. Figure S3(a) illustrates the beam splitting process for LCP light incident at arbitrary angles, with the co-polarized and cross-polarized transmission coefficients being $t_{LL}e^{i\phi_{LL}}$ and $t_{LR}e^{i\phi_{LR}}$, respectively. Figure S3(b) shows the variation in transmission efficiency as the incident angle changes from $-15°$ to $+15°$. It can be observed that within the calculated angle range, the transmission efficiency exhibits a slight variation, with the cross-polarized conversion efficiency being approximately twice that of the co-polarized conversion efficiency, while the total transmission efficiency is close to 1. Figure S3(c) further demonstrates the change in transmission phase with respect to the incident angle, indicating a relatively small variation in transmission phase with angle. The difference between the two transmission phases keeps 270° approximately, with a slight variation. Figures S3(d)-(f) present the simulation results for RCP light incidence, which are similar to those for LCP light. This indicates that the four sets of parallel beam splitting processes selected in the main text have approximately the same beam splitting response.

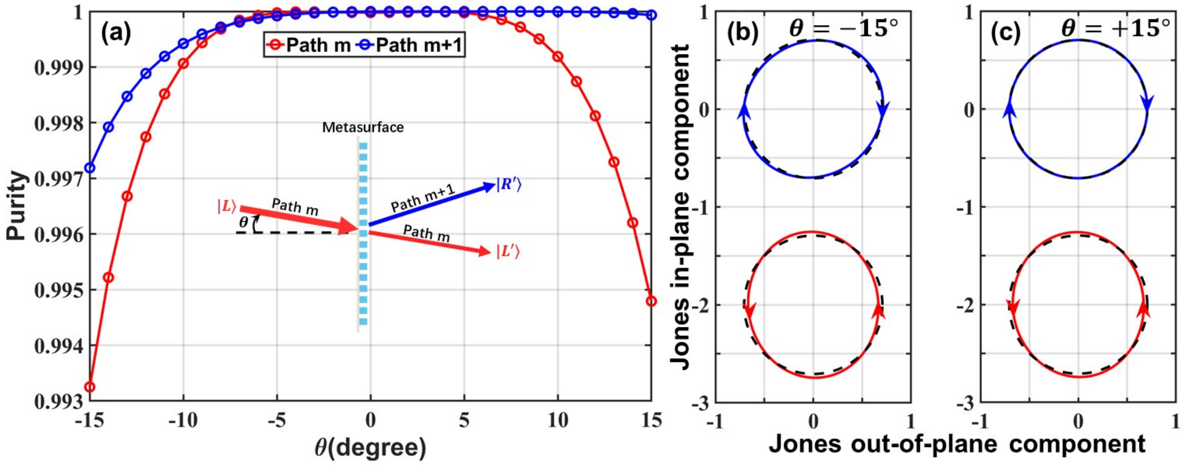

Figure S4. Angular dependence of circular polarization purity for the designed metasurface. (a) Variation of the circular polarization purity with the angle of incidence. The polarization ellipse of the output light at an incident angle of (b) $\theta = -15°$ and (c) $\theta = +15°$. The black dashed circles in (b) and (c) are the reference for perfect circularly polarized light.

Next, we analyze circular polarization purity of the output state and its dependence to the incident angle. Taking the incidence of LCP light as an example, as depicted in the inset of Fig. S4(a), under ideal conditions, the 0th-order transmitted light is perfect LCP,

and the +1th-order diffracted light is perfect RCP. The circular polarization purity of the output light is quantified by us using the absolute value of the degree of circular polarization (DoCP):

$$\text{Purity} = \left| \frac{|\langle E_R|E_{\text{out}}\rangle|^2 - |\langle E_L|E_{\text{out}}\rangle|^2}{|\langle E_R|E_{\text{out}}\rangle|^2 + |\langle E_L|E_{\text{out}}\rangle|^2} \right| \tag{S4}$$

where $|E_{L/R}\rangle$ represents the unit LCP/RCP Jones vector, and $|E_{\text{out}}\rangle$ represents the Jones vector of the output polarization state on a specific output path. The variation of the circular polarization purity of the two output lights with the incident angle is presented in Fig. S4(a). It can be seen that as the incident angle moves away from the normal direction, the purity starts to decline. However, for the metasurface we designed, the working angle is less than 15 degrees, at which the circular polarization purity of the two output lights stays above 99.3%. The polarization ellipses of the two output lights when the incident angle is ±15 degrees are further shown in Fig. S4(b)(c) (the black dashed line serves as the reference for perfect circularly polarized light), and it can be observed that the polarization ellipses are nearly perfect circles. Hence, within the working angle range under consideration, the circular polarization purity is almost 1, and the change in polarization state is negligible.

Through the above calculations, we can obtain the classical parallel beam splitting transformation relationship for the designed metasurface

$$\begin{pmatrix} |R_{\text{out}}\rangle_{+2} \\ |L_{\text{out}}\rangle_{+1} \\ |R_{\text{out}}\rangle_{+1} \\ |L_{\text{out}}\rangle_{0} \\ |R_{\text{out}}\rangle_{0} \\ |L_{\text{out}}\rangle_{-1} \\ |R_{\text{out}}\rangle_{-1} \\ |L_{\text{out}}\rangle_{-2} \end{pmatrix} \approx \begin{pmatrix} 0.56 & 0.81e^{1.48i} & 0 & 0 & 0 & 0 & 0 & 0 \\ 0.82e^{1.69i} & 0.56 & 0 & 0 & 0 & 0 & 0 & 0 \\ 0 & 0 & 0.57 & 0.81e^{1.47i} & 0 & 0 & 0 & 0 \\ 0 & 0 & 0.81e^{1.70i} & 0.57 & 0 & 0 & 0 & 0 \\ 0 & 0 & 0 & 0 & 0.57 & 0.81e^{1.46i} & 0 & 0 \\ 0 & 0 & 0 & 0 & 0.81e^{1.71i} & 0.57 & 0 & 0 \\ 0 & 0 & 0 & 0 & 0 & 0 & 0.56 & 0.82e^{1.45i} \\ 0 & 0 & 0 & 0 & 0 & 0 & 0.81e^{1.72i} & 0.56 \end{pmatrix} \begin{pmatrix} |R_{\text{in}}\rangle_{+2} \\ |L_{\text{in}}\rangle_{+1} \\ |R_{\text{in}}\rangle_{+1} \\ |L_{\text{in}}\rangle_{0} \\ |R_{\text{in}}\rangle_{0} \\ |L_{\text{in}}\rangle_{-1} \\ |R_{\text{in}}\rangle_{-1} \\ |L_{\text{in}}\rangle_{-2} \end{pmatrix} \tag{S5}$$

Note that $|L_{\text{in,out}}\rangle_j$ and $|R_{\text{in,out}}\rangle_j$ here represent the amplitudes of the LCP/RCP modes in path j, rather than Jones vectors. It is observed that the simulated parallel beam splitting transformation (S5) slightly deviates from the theoretical predictions provided in the main text. In the simulation results, the transformation matrix is not strictly symmetric, and the phase difference between the co-polarized and cross-polarized beams is not exactly π, but it

still approximates a unitary transformation.

S3. PERFORMANCES OF METASURFACES QUANTUM CZ GATE

In this section, we analyze the performance of quantum gates using the parallel beam splitting relationships obtained from metasurface simulations. Formally, the transformation matrix for quantum parallel beam splitting is the same as that of classical parallel beam splitting

$$\begin{pmatrix} \hat{V}^{R\dagger} \\ \hat{T}_0^{L\dagger} \\ \hat{T}_1^{R\dagger} \\ \hat{C}_1^{L\dagger} \\ \hat{C}_0^{R\dagger} \\ \hat{S}_1^{L\dagger} \\ \hat{S}_0^{R\dagger} \\ \hat{V}^{L\dagger} \end{pmatrix}_{\text{in}} \approx U^{\mathrm{T}} \begin{pmatrix} \hat{V}^{R\dagger} \\ \hat{T}_0^{L\dagger} \\ \hat{T}_1^{R\dagger} \\ \hat{C}_1^{L\dagger} \\ \hat{C}_0^{R\dagger} \\ \hat{S}_1^{L\dagger} \\ \hat{S}_0^{R\dagger} \\ \hat{V}^{L\dagger} \end{pmatrix}_{\text{out}} \tag{S6a}$$

$$U = \begin{pmatrix} 0.56 & 0.81e^{1.48i} & 0 & 0 & 0 & 0 & 0 & 0 \\ 0.82e^{1.69i} & 0.56 & 0 & 0 & 0 & 0 & 0 & 0 \\ 0 & 0 & 0.57 & 0.81e^{1.47i} & 0 & 0 & 0 & 0 \\ 0 & 0 & 0.81e^{1.70i} & 0.57 & 0 & 0 & 0 & 0 \\ 0 & 0 & 0 & 0 & 0.57 & 0.81e^{1.46i} & 0 & 0 \\ 0 & 0 & 0 & 0 & 0.81e^{1.71i} & 0.57 & 0 & 0 \\ 0 & 0 & 0 & 0 & 0 & 0 & 0.56 & 0.82e^{1.45i} \\ 0 & 0 & 0 & 0 & 0 & 0 & 0.81e^{1.72i} & 0.56 \end{pmatrix} \tag{S6b}$$

where the matrix U coincides with classical simulation results [equation (S5)], the superscript T indicates the transpose operation of matrices. Note that the relation in equation (S6) is the transformation from output modes to input modes, so that an input mode can be expressed as the superposition of output modes. But the equation (S5) describes the transformation from input modes to output modes. Another thing to note is that equation (S6) describes the quantum transformation for creation operators rather than for annihilation operators. With

above quantum transformation matrix, one can obtain the operation rules of our designed metasurface quantun gate [5].

We first consider the case of a single quantum CZ operation. The CZ operation is considered between the polarization qubits C (path 0) and T (path +1). The quantum logical operation between the two qubits is obtained by analyzing evolutions of four standard computation bases input:

$$
\begin{aligned}
|0\rangle_C|0\rangle_T = &\hat{C}_0^{R\dagger}\hat{T}_0^{L\dagger}|\varnothing\rangle_C|\varnothing\rangle_T \xrightarrow{\text{Metasurface}} (U_{55}\hat{C}_0^{R\dagger}+U_{65}\hat{S}_1^{L\dagger})(U_{22}\hat{T}_0^{L\dagger}+U_{12}\hat{V}^{R\dagger})|\varnothing\rangle_C|\varnothing\rangle_T \\
&\xrightarrow{\text{Postselection}} U_{22}U_{55}\hat{C}_0^{R\dagger}\hat{T}_0^{L\dagger}|\varnothing\varnothing\rangle = U_{22}U_{55}|0\rangle_C|0\rangle_T \qquad \text{(S7a)}\\
|0\rangle_C|1\rangle_T = &\hat{C}_0^{R\dagger}\hat{T}_1^{R\dagger}|\varnothing\rangle_C|\varnothing\rangle_T \xrightarrow{\text{Metasurface}} (U_{55}\hat{C}_0^{R\dagger}+U_{65}\hat{S}_1^{L\dagger})(U_{33}\hat{T}_1^{R\dagger}+U_{43}\hat{C}_1^{L\dagger})|\varnothing\rangle_C|\varnothing\rangle_T \\
&\xrightarrow{\text{Postselection}} U_{33}U_{55}\hat{C}_0^{R\dagger}\hat{T}_1^{R\dagger}|\varnothing\rangle_C|\varnothing\rangle_T = U_{33}U_{55}|0\rangle_C|1\rangle_T \qquad \text{(S7b)}\\
|1\rangle_C|0\rangle_T = &\hat{C}_1^{L\dagger}\hat{T}_0^{L\dagger}|\varnothing\rangle_C|\varnothing\rangle_T \xrightarrow{\text{Metasurface}} (U_{44}\hat{C}_1^{L\dagger}+U_{34}\hat{T}_1^{R\dagger})(U_{22}\hat{T}_0^{L\dagger}+U_{12}\hat{V}^{R\dagger})|\varnothing\rangle_C|\varnothing\rangle_T \\
&\xrightarrow{\text{Postselection}} U_{22}U_{44}\hat{C}_1^{L\dagger}\hat{T}_0^{L\dagger}|\varnothing\rangle_C|\varnothing\rangle_T = U_{22}U_{44}|1\rangle_C|0\rangle_T \qquad \text{(S7c)}\\
|1\rangle_C|1\rangle_T = &\hat{C}_1^{L\dagger}\hat{T}_1^{R\dagger}|\varnothing\rangle_C|\varnothing\rangle_T \xrightarrow{\text{Metasurface}} (U_{44}\hat{C}_1^{L\dagger}+U_{34}\hat{T}_1^{R\dagger})(U_{33}\hat{T}_1^{R\dagger}+U_{43}\hat{C}_1^{L\dagger})|\varnothing\rangle_C|\varnothing\rangle_T \\
&\xrightarrow{\text{Postselection}} (U_{33}U_{44}+U_{34}U_{43})\hat{C}_1^{L\dagger}\hat{T}_0^{L\dagger}|\varnothing\rangle_C|\varnothing\rangle_T = (U_{33}U_{44}+U_{34}U_{43})|1\rangle_C|0\rangle_T \\
& \qquad \text{(S7d)}
\end{aligned}
$$

where $|\varnothing\rangle$ denotes the quantum vacuum state, and the zero element in matrix U is eliminated. Through postselection, the output state remains a two-qubit system by retaining only components where a single photon occupies each logical-encoding mode. Then, the metasurface's transformation results for the four 2-qubit standard bases are as shown in Tab. S1(a). It can be observed that the standard bases still transform to the same standard bases. Notably, there are some deviations from the theoretical truth table presented in the main text. On one hand, when both qubits are in the logical 1 state, the output state acquires an additional phase of 1.018π instead of the ideal π. On the other hand, the success probability of the operation has undergone a slight change. The success probabilities for the four cases are not strictly equal (but still approximately so), with the success probability being slightly less than the theoretical value of $1/9 \approx 0.111$. This is due to the beam splitting ratio of the designed metasurface not being strictly 2:1, and there is a slight reflection from the metasurface. Similarly, the truth table for the quantum operation between the polarization qubits C (path 0) and S (path -1) under parallel operation can be obtained, shown in Tab. S1(b). Due to the special encoding rules presented in the main text, the qubit

TABLE S1. Transformations of two-qubit bases under the quantum operation of the metasurface. (a) Truth table of quantum operation between qubits C and T. (b) Truth table of quantum operation between qubits C and S.

(a)

Input	Output	Success probability
$\|0\rangle_C\|0\rangle_T$	$\|0\rangle_C\|0\rangle_T$	0.102
$\|0\rangle_C\|1\rangle_T$	$\|0\rangle_C\|1\rangle_T$	0.106
$\|1\rangle_C\|0\rangle_T$	$\|1\rangle_C\|0\rangle_T$	0.102
$\|1\rangle_C\|1\rangle_T$	$e^{1.018\pi i}\|1\rangle_C\|1\rangle_T$	0.110

(b)

Input	Output	Success probability
$\|0\rangle_C\|0\rangle_S$	$\|0\rangle_C\|0\rangle_T$	0.102
$\|0\rangle_C\|1\rangle_S$	$e^{1.018\pi i}\|0\rangle_C\|1\rangle_T$	0.110
$\|1\rangle_C\|0\rangle_S$	$\|1\rangle_C\|0\rangle_T$	0.102
$\|1\rangle_C\|1\rangle_S$	$\|1\rangle_C\|1\rangle_T$	0.106

S is controlled by logical state 0 of qubit C, i.e., an additional phase of 1.018π arises when the input state is $|0\rangle_C|1\rangle_S$. If one exchanges the logical encoding states of qubit C, then the quantum operation between qubits C and S is a normal CZ operation approximately.

Next, we examine the situation when the two quantum CZ gates mentioned above are cascaded. Utilizing the parallel beam splitting transformation rules of the metasurface, we transform each of the 8 three-qubit standard bases, with the results shown in Tab. S2. Similar to the results for a single CZ gate, the terms that should ideally accumulate a phase π now accumulate a phase of 1.018π. The success probability of the cascaded operation is no longer strictly equal but remains approximately so, with the success probability slightly reduced due to imperfect beam splitting ratios and reflections (ideal value is $1/27 \approx 0.037$).

Lastly, we consider the performance of the designed metasurface CZ gate in the preparation of entangled states. For the preparation of a two-qubit entangled state, considering the input as the two-qubit direct product state $|+\rangle_C|+\rangle_T$, where $|\pm\rangle = (|0\rangle \pm |1\rangle)/\sqrt{2}$. According to the truth table provided above, the metasurface transformation the input state

TABLE S2. Characteristic of a two-cascaded CZ gate on designed metasurface.

Input	Output	Success probability
$\|0\rangle_C\|0\rangle_S\|0\rangle_T$	$\|0\rangle_C\|0\rangle_S\|0\rangle_T$	0.032
$\|0\rangle_C\|0\rangle_S\|1\rangle_T$	$\|0\rangle_C\|0\rangle_S\|1\rangle_T$	0.033
$\|0\rangle_C\|1\rangle_S\|0\rangle_T$	$e^{1.018\pi i}\|0\rangle_C\|1\rangle_S\|0\rangle_T$	0.035
$\|0\rangle_C\|1\rangle_S\|1\rangle_T$	$e^{1.018\pi i}\|0\rangle_C\|1\rangle_S\|1\rangle_T$	0.036
$\|1\rangle_C\|0\rangle_S\|0\rangle_T$	$\|1\rangle_C\|0\rangle_S\|0\rangle_T$	0.032
$\|1\rangle_C\|0\rangle_S\|1\rangle_T$	$e^{1.018\pi i}\|1\rangle_C\|0\rangle_S\|1\rangle_T$	0.035
$\|1\rangle_C\|1\rangle_S\|0\rangle_T$	$\|1\rangle_C\|1\rangle_S\|0\rangle_T$	0.033
$\|1\rangle_C\|1\rangle_S\|1\rangle_T$	$e^{1.018\pi i}\|1\rangle_C\|1\rangle_S\|1\rangle_T$	0.036

into

$$|\psi_1\rangle = 0.228|0\rangle_C|+\rangle_T + (0.230 + 0.007i)|1\rangle_C|-\rangle_T + |\psi_1\rangle_{\text{error}} \tag{S8a}$$

$$|\psi_1\rangle_{\text{error}} = -0.002|0\rangle_C|-\rangle_T - (0.004 + 0.007i)|1\rangle_C|+\rangle_T \tag{S8b}$$

Under a perfect quantum CZ operation, the output state should be a Bell-type state $|\psi_{\text{Bell}}\rangle = (|0\rangle_C|+\rangle_T + |1\rangle_C|-\rangle_T)/3\sqrt{2}$. It can be seen that, due to the imperfect beam splitting of the metasurface, the output state after the metasurface CZ gate transformation is not a perfect Bell-type entangled state, and an additional error term $|\psi_1\rangle_{\text{error}}$ appears, although the error component is very small. The fidelity of the output state is

$$F_1 = \frac{|\langle\psi_{\text{Bell}}|\psi_1\rangle|^2}{\langle\psi_{\text{Bell}}|\psi_{\text{Bell}}\rangle\langle\psi_1|\psi_1\rangle} \approx 0.9992 \tag{S9}$$

The fidelity is close to 1, indicating that the single quantum CZ gate on the metasurface has a high operational fidelity.

For the preparation of a three-qubit entangled state, considering the input as the three-qubit direct product state $|+\rangle_C|+\rangle_S|+\rangle_T$. The designed metasurface transformation the input state into

$$|\psi_2\rangle = 0.130e^{0.029i}(|0\rangle_C|-\rangle_S|+\rangle_T + |1\rangle_C|+\rangle_S|-\rangle_T) + |\psi_2\rangle_{\text{error}} \tag{S10a}$$

$$\begin{aligned}|\psi_2\rangle_{\text{error}} = {}& 0.004e^{-2.132i}(|0\rangle_C|+\rangle_S|+\rangle_T + |1\rangle_C|+\rangle_S|+\rangle_T)\\ &+ 4\times10^{-5}e^{1.009i}(|0\rangle_C|+\rangle_S|-\rangle_T + |1\rangle_C|-\rangle_S|+\rangle_T)\\ &+ 0.001e^{-3.113i}(|0\rangle_C|-\rangle_S|-\rangle_T + |1\rangle_C|-\rangle_S|-\rangle_T)\end{aligned} \tag{S10b}$$

Also, a small error term $|\psi_2\rangle_{\text{error}}$ arises. Under a perfect cascade quantum CZ operation as shown in the main text, the output state should be a GHZ-type state $|\psi_{\text{GHZ}}\rangle = (|0\rangle_C|-\rangle_S|+\rangle_T + |1\rangle_C|+\rangle_S|-\rangle_T)/3\sqrt{6}$. Then the fidelity of the output state is

$$F_2 = \frac{|\langle\psi_{\text{GHZ}}|\psi_2\rangle|^2}{\langle\psi_{\text{GHZ}}|\psi_{\text{GHZ}}\rangle\langle\psi_2|\psi_2\rangle} \approx 0.9988 \tag{S11}$$

The high fidelities of the prepared entangled states (F_1, F_2) demonstrate that both the designed metasurface quantum CZ gate and the cascaded CZ gates possess high operational fidelity.

[1] Arbabi, A. et al. Dielectric metasurfaces for complete control of phase and polarization with subwavelength spatial resolution and high transmission. *Nature Nanotechnology.* **10**, 937–943 (2015).

[2] Balthasar Mueller, J. P. et al. Metasurface polarization optics: independent phase control of arbitrary orthogonal states of polarization. *Physical Review Letters* **118**, 113901 (2017).

[3] Georgi, P. et al. Metasurface interferometry toward quantum sensors. *Light: Science & Applications* **8**, 70 (2019).

[4] Hugonin, J. P. & Lalanne, P. RETICOLO software for grating analysis. *arXiv* :2101.00901 (2021).

[5] Ralph, T. C. et al. Linear optical controlled-Not gate in the coincidence basis. *Physical Review A* **65**, 062324 (2002).
